# Effect of Initial Position and Crystallographic Orientation on Grain Selection Procedure in Z-Form Selector for Ni-Based Single-Crystal Superalloy

**DOI:** 10.3390/ma17081885

**Published:** 2024-04-19

**Authors:** Yuanyuan Guo, Jun Bao, Xuanning Zhang, Mai Zhang, Xiqiong Yang, Jian Zhang

**Affiliations:** 1 Science and Technology on Advanced High Temperature Structural Materials Laboratory, AECC Beijing Institute of Aeronautical Materials, Beijing 100095, China; guoyuanyuanjida@163.com (Y.G.); zhangxuanning2018@163.com (X.Z.); 18811317156@163.com (M.Z.);; 2 AECC Commercial Aircraft Engine Co., Ltd., Shanghai 200241, China

**Keywords:** Ni-based single-crystal superalloy, grain selection process, competitive growth, temperature field, crystal orientation

## Abstract

The grain selection process in a Z-form selector for Ni-based single-crystal superalloy was simulated using a macro-scale ProCAST software (2013 version) coupled CAFE module combined with an experiment to investigate the grain selection procedure and mechanism with different grain positions and crystal orientation relationships. A non-stationary solidification process was found in the Z-form selector, and the liquid–solid (L–S) interface was tilted in the same direction as the selector channel during directional solidification. Given that the grain boundary was parallel to the Z-form selector, the overgrowth rate of the bi-crystal in the selector channel was very low. The initial position of the bi-crystal in the selector channel has a greater effect on the overgrowth rate than the effects of primary and secondary orientations. The grain selection was a result of the coupling of the competitive grain growth effect and geometrical restriction effect. Finally, the selection grain mechanism within the Z-form selector was discussed, coalescing the temperature field and the grain competition growth mechanism.

## 1. Introduction

Nickel-based single-crystal, high-temperature alloys are widely used as aero-engine turbine blades due to their excellent high-temperature strength and creep resistance [1]. The service performance of single-crystal turbine blades is quite sensitive to crystallographic orientation [2,3,4]. In industrial production, the seeding or grain selection technique is the common method used in the casting process to control the crystallographic orientation of the single-crystal component [5,6,7,8,9]. The nature of the grain selection method is that a large number of randomly oriented grain grow competitively to obtain only one grain within a grain selector, which usually consists of a starter block and a selector [10,11,12]. Compared to the seeding method, the grain selection method has the advantages of high efficiency and low expense, which makes it widely used in industrial production.

In order to improve the control accuracy of the crystallographic orientation of single-crystal blades, the researchers paid in-depth attention to the grain selection process and its mechanism [9,12,13,14,15,16,17]. The main role of the starter block in the grain selection process is widely considered to be the acquisition of <001> oriented grain clusters [18,19]. However, the main role of a selector and its grain selection mechanism are still unclear [9,13,16,19,20]. Dai et al. found that the crystallographic orientation of surviving grain cannot be optimized and, thus, proposed that the geometrical restriction effect is the primary mechanism governing the grain selection procedure in the selector [19,21]. Meng et al. studied the process of selecting grain in the selector with various geometries and sizes and showed that the crystallographic orientation of the surviving grain could be further optimized in the selector [22]. Using a two-dimensional grain selector, Zhu et al. found that the grain selection efficiency largely depends on the selector wire diameter and the take-off angle [15]. According to research by Seo et al., the grain that is closest to the inner wall of the selector frequently develops high-branching dendrites to overgrow other grains and is subsequently chosen as the surviving grain [16]. Combining experimental and numerical simulation results, Wang et al. suggested that the combined effects of space expansion, geometrical restriction and grain competition govern the grain selection procedure in the selector [13]. They further found that the contribution of secondary dendrites to overgrowth between grains in the selector is greater than that of primary dendrites, and, therefore, the selector cannot optimize the crystallographic orientation of the surviving grain. However, it is not clear which initial feature of the grain just entering the selector channel, such as grain position, primary or secondary crystallographic orientation, is the key factor controlling the behavior of grain selection.

A generally accepted model of competitive grain growth was proposed by Walton and Chalmers in 1959 and schematically summarized by Rappaz in 1994, which was known as W–C model [23,24]. Since then, several experiments and simulations have revealed some phenomena that cannot be explained by the model [25,26,27,28]. However, a large number of experiments have confirmed the high accuracy of the grain selection behavior within various selectors predicted by numerical simulation using ProCAST simulation combined with CAFE module, a mesoscopic microstructure simulation module developed based on the W–C model [16,18,19,21,22]. Currently, ProCAST simulation in combination with the CAFE module has become one of the most important methods to study the grain selection mechanism and to optimize the geometry and size of the selector.

Therefore, directional solidification experiments and numerical simulations using ProCAST software (2013 version) combined with the CAFE module were carried out to study the temperature field evolution and the competitive grain growth during directional solidification in a simplified 2D Z-form selector. By carefully regulating the initial characteristics of the grains entering the selector, the influence and mechanism of the initial position and crystallographic orientation on grain selection behavior were examined in this research. These efforts may provide new insights into the clarification of the grain selection mechanism in the selector.

## 2. Materials and Methods

**Figure 1:**
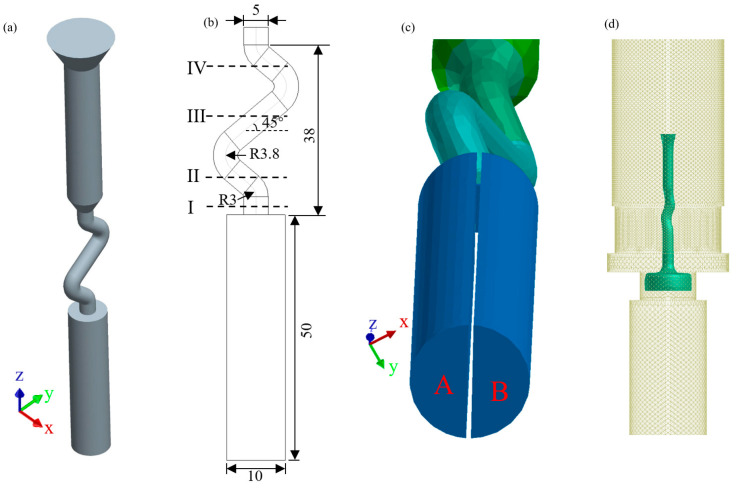
The model of the directional solidification; (**a**) the coordinate relationship of the casting in the simulation system; (**b**) the dimensions of the 2D selector; (**c**) the angle between the thin slice and YOZ plane is 30°; and (**d**) the FEM mesh for simulation.

The material used in this study was a second-generation, single-crystal superalloy DD6, and its normal composition (wt.%) was Cr4.3, Co9.0, Mo2.0, W8.0, Ta7.5, Re2.0, Hf0.1, Al5.6, Nb0.5, and Ni Bal. The directional solidification experiment was realized used a bi-seeding method under a modified Bridgman directional casting furnace. The casting structure of the 2D Z-form selector is shown in Figure 1a; meanwhile, Figure 1b displays the dimensions of the selector. The bi-seeds were placed in mold according to the position characteristics shown in Figure 1c. It can be seen that the selector channel of the 2D selector is parallel to the YOZ plane. The crystallographic orientation of the bi-crystal was depicted used Euler angle (*Φ1*, *Φ*, *Φ2*). With the coordinate system shown in Figure 1a, the crystallographic orientations of grains A and B were 0,0,0 and 0,20,0, respectively. During directional solidification, the mold was heated to 1550 °C. After the ingot was melted, the liquid melt was poured into the preheated mold cavity and held for 10 min to stabilize. Finally, the mold was withdrawn from the furnace at a pre-determined withdrawal rate of 50 μm/s. Following directional solidification, samples were subsequently machined, polished, and etched with a mixture of HNO_3_, HF, and C_3_H_8_O_3_ (volume ratio 1:2:3) and then observed with an optical microscope (DM-4000M; Leica, Berlin, Germany).

The directional solidification process was simulated using the ProCAST software (a trademark of ESI Group, Paris, France) coupled with a cellular automaton finite element (CAFE) module. Figure 1d depicts the finite element mesh (FEM) model of a directional solidification system. Solid 3D meshes were used to construct the casting and ceramic mold. To improve calculation efficiency, the furnace model was constructed with a 2D boundary mesh, and the withdrawal process was accomplished through the upward movement of the furnace. Using the temperature profiles obtained from the ProCAST simulation as input data, the CAFE module was then carried out to predict the grain structure with specific nucleation and growth kinetic parameters. The parameters used in this simulation were derived from the literature [18], because the study successfully predicts the grain selection process in the selector, which is in relatively good agreement with the experimental results. And some of key parameters are shown in Table 1.

As shown in Figure 1b, a thin slice is cut off along the axis of the starter block, dividing it evenly into two sections. During the CAFE simulation, the bottom surfaces A and B of the starter block were configured to the surface nucleation (single crystal type, Euler angle) to precisely regulate the crystallographic orientation of the grains entering the selector. The angle between the thin slice and the YOZ plane controls the position characteristics of the grains as they enter the selector. The angles between the thin slice and the YOZ plane and corresponding surface nucleation (single crystal type) at the bottom surface used in this work are shown in Table 2.

## 3. Results

**Figure 2:**
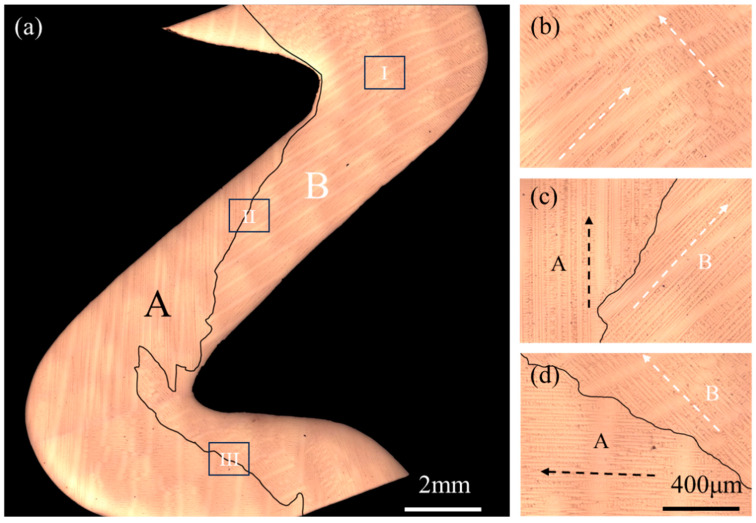
The longitudinal section microstructure of bi-crystal in the Z-form selector (a) and local magnification (**b**) I region, (**c**) II region, and (**d**) III region. The black solid line marks the location of the grain boundary, and the dendrite growth direction is marked by the virtual arrow.

Figure 2a showed the longitudinal section microstructure of the bi-crystal in the Z-form selector, and the black solid line marked the location of the grain boundary. It could be seen that the grain boundary of the bi-crystal was tilted towards the grain A when the bi-crystal just entered the Z-form selector, indicating that the grain B was dominant in the competitive grain growth. However, the grain B overall presented an eliminated growth tendency due to the strong geometrical restriction effect. After the first turn point of the Z-form selector, the growth behavior of the bi-crystal showed completely different characteristics from that with the initial stage. Grain A prevailed during the competitive grain growth process, but it was also subjected to a severe geometrical restriction effect. Therefore, the grain B presented showed a tendency to grow up in this region due to the combined effects of competitive growth and geometrical restriction. The growth tendency of the bi-crystal showed the initial character again after the next turning point of the selector. Finally, the single crystal was not obtained after passing through the Z-form selector due to the continuous transformation in the growth trend of the bi-crystal.

To further display the competitive growth process, local magnification within the different regions are shown in Figure 2b–d, and the dendrite growth direction was marked by virtual arrow. It could be seen that the primary dendrite growth direction of the bi-crystal changed after the grain growth reached the turning point of the selector, which was the main reason for the transformation in the competitive grain growth. According to the symmetry of the Ni-based single-crystal superalloy, the deviation angle of the primary dendrite growth direction and the heat flow direction was less than 45°. Therefore, the heat flow direction during bi-crystal competitive growth process in Z-form selector was located in the angle area of dendrite growth, which was basically the same as the direction of the selector channel.

**Figure 3:**
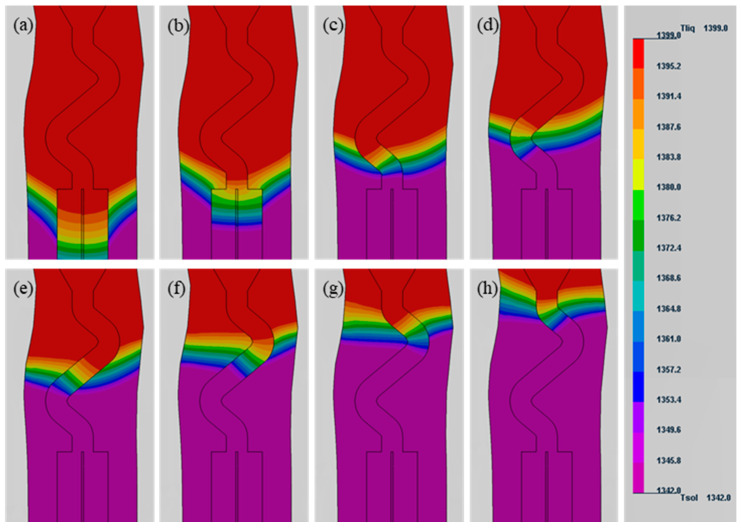
The temperature profiles of selector with various withdrawal time. (**a**) 329 s, (**b**) 422 s, (**c**) 492 s, (**d**) 542 s, (**e**) 592 s, (**f**) 642 s, (**g**) 692 s, and (**h**) 742 s.

The evolution of the temperature profiles (1342–1399 °C) of the selector during the directional solidification process is depicted in Figure 3. During the directional solidification process, the temperature field evolution was shown to be non-stationary. The liquid–solid (L–S) interface had a significantly depressed morphology, as illustrated in Figure 3a, since the solidification interface front was located in the starter block. The curvature of the L–S interface reduced as the solidification interface front moved to the selector channel. At the same time, the normal direction of the L–S interface was essentially consistent with the axis of the selector channel during solidification interface advancement. This was consistent with earlier findings on temperature field evolution in the three-dimensional grain selection process [13,18]. The temperature gradient (G) at the solid–liquid interface front during directional solidification can be approximated using the following equation:

G = ΔT/L

where ΔT is the solid–liquid phase temperature interval, and L is the length of the solid–liquid phase zone. For alloy DD6, the ΔT is a constant value of 57 °C. Therefore, the temperature gradient decreases with the increase in the length of the solid–liquid phase zone. It could also be noticed that the temperature gradient (53–114 °C/cm) in the selector channel displayed a tendency of growing and then reducing during directional solidification.

**Figure 4:**
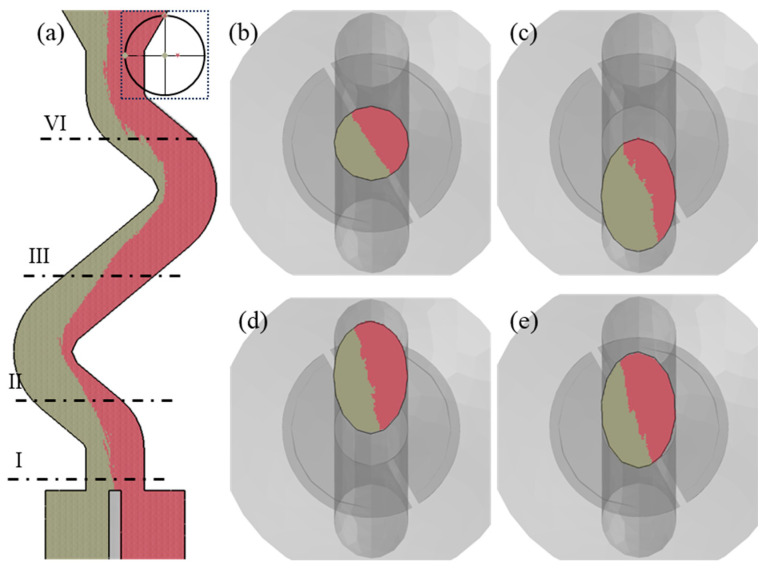
Grain selection process with 30° angle between the thin slice and the YOZ plane. (**a**) Grain selection process displayed in the longitudinal section and transverse cross section of (**b**) I, (**c**) II, (**d**) III, and (**e**) IV. The Euler angles of grain at bottom surface A and B were 0°, 0°, 0°, and 0°, 20°, 0°, respectively. The inset in (**a**) was the crystallographic orientation of bi-crystal shown in the polar figure.

In order to further verify that the grain selection process can be accurately simulated using ProCAST software coupled with the CAFE module, the simulation consistent with the experimental parameters was carried out, and the result is shown in Figure 4. It could be seen that the grain selection process in selector channel was basically consistent with the experiment results (shown in Figure 2a). From Figure 4b–e, the grain boundary was gradually parallel to the YOZ plane during the competitive bi-crystal growth process in the selector channel. 

**Figure 5:**
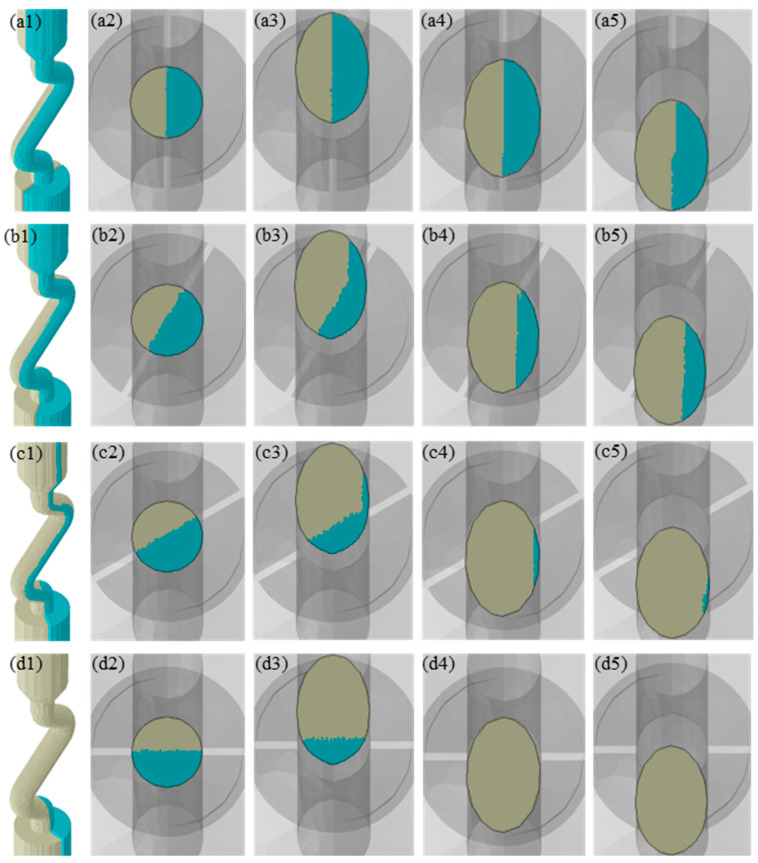
Grain selection process with different angles between the thin slice and the YOZ plane. The angles were (**a1**–**a5**) 0°, (**b1**–**b5**) 30°, (**c1**–**c5**) 60°, and (**d1**–**d5**) 90°. Grain selection process displayed in 3D (**a1**–**d1**) and transverse cross section of (**a2**–**d2**) I, (**a3**–**d3**) II, (**a4**–**d4**) III, and (**a5**–**d5**) IV. The Euler angles of grain at bottom surface A and B were 0°, 0°, 0°, and 0°, 5°, 0°, respectively.

Figure 5 depicted the results of the CAFE simulations conducted at different angles between the thin slice and the YOZ plane. It was clear that the initial grain boundary position of the bi-crystal at the entrance of the selector channel corresponded to the position of the thin slice. The size of the double grains remained relatively constant throughout the directional solidification process, as shown in Figure 4a, indicating that the bi-crystal may survive in a stable state under these conditions. The overgrowth rate of the bi-crystal rose as the angle between the grain boundary and the plane where the selector channel was situated increased, as illustrated in Figure 4b–d. It is north noting that, once the grain boundary was aligned with the plane where the selector channel was situated during the directional solidification, the overgrowth rate of bi-crystal in the competitive grain growth process within the selector channel was effectively decreased to zero. This implied that the selector had no grain selection effect, since the initial grain boundary of the bi-crystal was parallel to the plane where the selector channel was situated.

**Figure 6:**
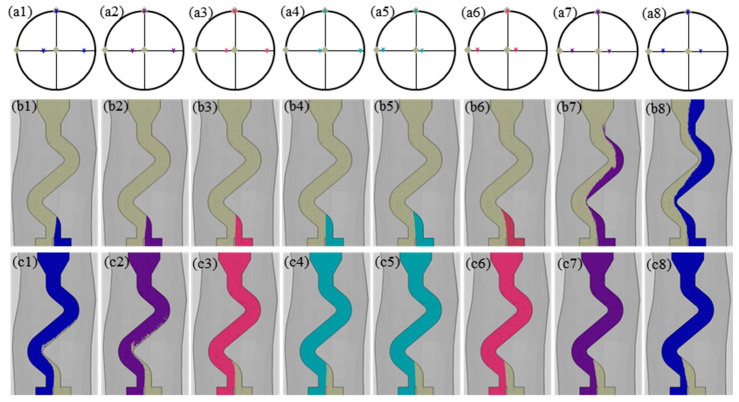
Grain selection process of bi-crystal with various primary orientation; (**a1**–**a8**) the crystallographic orientation of bi-crystal shown in the polar figure; (**b1**–**b8**) grain selection process with the favorably oriented grain located in an area with more growth space; (**c1**–**c8**) grain selection process with the unfavorably oriented grain located in an area with more growth space. The Euler angles of favorably oriented grains were 0°, 0°, and 0°; the Euler angles of unfavorably oriented grains were (**a1**–**c1**) 0°, −20°, 0°, (**a2**–**c2**) 0°, −15°, 0°, (**a3**–**c3**) 0°, −10°, 0°, (**a4**–**c4**) 0°, −5°, 0°, (**a5**–**c5**) 0°, 5°, 0°, (**a6**–**c6**) 0°, 10°, 0°, (**a7**–**c7**) 0°, 15°, 0°, (**a8**–**c8**) 0°, 20°, and 0°.

For a 90° angle between the grain boundary and the YOZ plane, the grain selection behavior of grains with a different primary orientation in the Z-form selector was explored, and the results are shown in Figure 6. The influence of the primary orientation on grain selection behavior was not significant when the <001> direction of the unfavorably oriented grains differed from the directional solidification (vertically upward) by less than 15°. The grain with the most growth space wins the competitive growth process once the bi-crystal enters the selector channel. The competitive growth behavior of the bi-crystal within the selector channel was connected to the primary orientation and initial position of the grains when the <001> direction of the unfavorably oriented grains diverged from the directional solidification by more than 15°. When placed in an area with more growth space, the unfavorably oriented grains could overgrow the favorably oriented grain. When unfavorably oriented grains were located in an unfavorable position, they were quickly eliminated when the <001> direction of the unfavorably oriented grains coincided with the direction of the selector channel, and, conversely, it could compete for growth with the favorably oriented grains in the long term.

**Figure 7:**
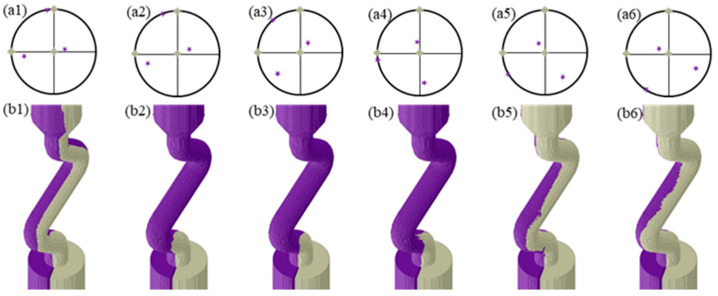
Grain selection process of bi-crystal with various secondary orientation of unfavorably oriented grains thorough regulating *Φ1*; (**a1**–**a6**) the crystallographic orientation of bi-crystal shown in the polar figure; (**b1**–**b6**) grain selection process. The Euler angles of favorably oriented grains were 0°, 0°, and 0°; the Euler angles of unfavorably oriented grains were (**a1**–**b1**) 5°, 15°, 0°, (**a2**–**b2**) 15°, 15°, 0°, (**a3**–**b3**) 45°, 15°, 0°, (**a4**–**b4**) 95°, 15°, 0°, (**a5**–**b5**) 120°, 15°, 0°, (**a6**–**b6**) 150°, 15°, and 0°.

**Figure 8:**
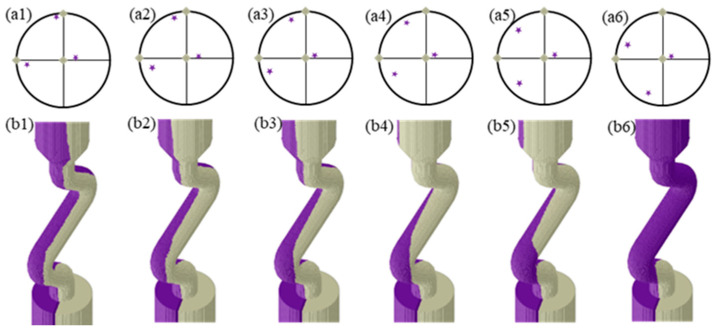
Grain selection process of bi-crystal with various secondary orientation of unfavorably oriented grains thorough regulating *Φ2*; (**a1**–**a6**) the crystallographic orientation of bi-crystal shown in the polar figure; (**b1**–**b6**) grain selection process. The Euler angles of favorably oriented grains were 0°, 0°, 0°; the Euler angles of unfavorably oriented grains were (**a1**–**b1**) 5°, 15°, 5°, (**a2**–**b2**) 5°, 15°, 10°, (**a3**–**b3**) 5°, 15°, 15°, (**a4**–**b4**) 5°, 15°, 25°, (**a5**–**b5**) 5°, 15°, 45°, (**a6**–**b6**) 5°, 15°, and 60°.

The grain selection behavior of grains with varied secondary orientations in the Z-form selector was investigated at a 30° angle between the grain boundary and the YOZ plane by regulating *Φ1* and *Φ2*, and the results are shown in Figure 7 and Figure 8, respectively. As demonstrated in Figure 6, the primary orientation of the unfavorably oriented grains remained constant, while the angle of <001> departure from the YOZ plane changed with the adjustment of *Φ1*, resulting in significant differences in the competitive growth process of the bi-crystal within the selection channel. Only when *Φ1* was between 45° and 120° did the unfavorably oriented grains succeed in the competitive growth process. Otherwise, both grains grew out of the selector channel, indicating a failure in the grain selection process. As shown in Figure 7, the primary orientation of the unfavorably oriented grains and angle of <001> departure from the YOZ plane changed with the adjustment of *Φ2*, while the crystallographic direction rotated along the <001> direction. Only when *Φ2* was 120° did the unfavorably oriented grains win in the competitive growth process. Otherwise, the grain selection process failed.

## 4. Discussion

Because of the high anisotropy of the Ni-based single crystal superalloy, accurate control of the crystallographic orientation of the blades has become one of the most essential ways for improving service performance. As a result, a significant effort has been expended on the grain selection process and optimization in order to increase the control of precision on the crystallographic orientation achieved by the grain selection method [6,10,18,29,30,31,32]. Researchers have clarified the essence of grain selection behavior through in-depth research on competitive grain growth and grain selection behavior, and the main role of the starter block is to obtain the <001> oriented texture through the competitive growth of a large number of grains [13,16,19]. However, there is no agreement on the main role of the selector and its mechanism. 

The grain selection behavior is mostly a competitive grain growth process within a specific component [9]. However, the grain selection process differs from the general process of competitive grain growth and exhibits distinctive characteristics [9,14,15,23,24,25]. To begin, the structure of the selector is complex. The spiral selector is the most frequent type of selector during industrial production, but there are various other types of selectors, such as restrictor, angled, and so on [1]. They all have narrow selection channels and complex structures in common. Seo et al. discovered that the grain towards the inner wall of the selector channel is typically chosen as the final single crystal [16]. According to this phenomenon, Gao et al. concluded that the geometric restriction effect is mostly responsible for the grain selection process [19]. Recently, Wang et al. further found that the grain which can continually expand out of higher dendrites becomes the final single crystal, and, thus, proposed that the combination effect with space expansion, geometrical restriction, and competitive grain growth is the key mechanism of grain selection in the selector [13]. As shown in Figure 5, in the initial stage of the grain selection process, the grain boundary of the be-crystal is aligned with the direction of the selector channel. The grain located in the more expanded space can overgrow the grain whose growth space is blocked during the competitive growth process within the selector, where the geometric restriction mainly acts on the eliminated grain, and the space expansion effect mainly acts on the final single crystal. When the grain boundary is parallel to the YOZ plane, the geometric restriction and space expansion effect both act on the double grain to the same extent so that they coexist in a steady state within the selector channel, leading to the failure of grain selection. This indicates that the grains are subjected to varying degrees of geometric limitation and the space expansion effect coupling as the primary mechanism of grain selection within the selector channel. 

Secondly, the evolution of the temperature field was featured within the selector. Due to the narrow and complex structure of the selector and its proximity to the water-cooling plate, the heat flow was mainly dominated by conduction along the solidified superalloy during directional solidification, resulting in the normal L–S interface basically being parallel to the selector channel, as shown in Figure 3. The shape and slop of the L–S interface affect the dendrite growth and grain selection behavior. Miller et al. found that the onset of lateral growth of secondary dendrite arms over the primary arm occurred as the L–S interface was inclined by at least 45° [33]. The angle between the selector channel and the directional solidification was usually between 20° and 70°, mainly concentrated between 45° and 65° [18,22]. Therefore, the branching behavior of dendrite in the selector channel must be affected by the L–S interface, which in turn has a significant effect on the grain selection behavior. 

**Figure 9:**
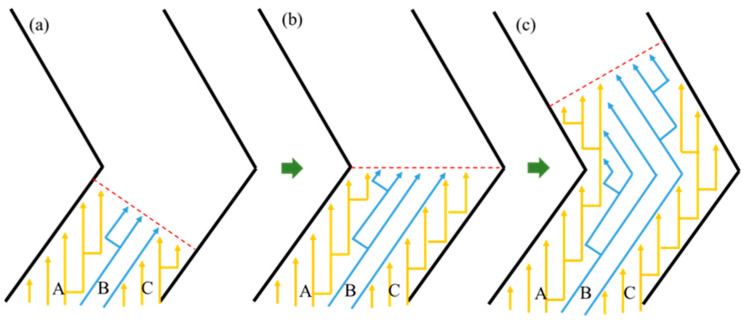
A schematic diagram of the microstructure evolution of grains within selector at different stage; (**a**) the L–S interface initially enters the selector; (**b**) the L–S interface advances to the turn of the selector; (**c**) the L–S interface is, again, advanced to the tilt of the selector.

A schematic diagram of the microstructure evolution of the grains within the selector is shown in Figure 9. During the directional solidification, the dendrite tip is slightly behind the L–S interface, which is represented by the red dashed line as shown in Figure 9. The overgrowth rate of competitive grain growth within the selector channel is accelerated by the combined effect of space expansion and geometric restriction, which also has an important influence on the overgrowth results. As shown in Figure 9a, the grain A experiences geometric restriction during competitive growth with grain B, resulting in a steady contraction of the growth space. Because of the space expansion effect, grain C can continue to rise upward in the competitive growth process. At the same time, due to the advantage of the crystallographic orientation, grain B can also win in the competitive growth process, resulting in a progressive expansion in the growth space. The branching behavior of the dendrite changes as the direction of the selector channel changes due to the action of the L–S interface. The principal branching direction is always less than 45° from the normal direction of the L–S interface, as seen in Figure 9a–c. Due to the space expansion effect, grain A expands gradually during the grain competition growth process, whereas grains B and C are eliminated during the competition growth process due to the geometric restriction effect. The effect of crystallographic orientation on competitive grain growth has received a lot of attention [24,25,27]. The primary dendritic orientation controls the type of competitive growth, whereas the secondary orientation influences dendrite branching behavior at grain boundaries. As a result, primary orientation has a bigger impact on competitive growth outcomes and overgrowth rates during conventional competitive grain growth than the secondary orientation. When viewed in conjunction with Figure 5, Figure 6 and Figure 7, it is clear that changing both the primary and secondary dendritic orientations have a bigger effect on grain selection behavior than changing simply the primary or secondary orientations.

## 5. Conclusions

The grain selection process in the Z-selector of a nickel-based single-crystal superalloy was simulated using the macro-scale ProCAST software coupled with the CAFE module. During the directional solidification process, the liquid–solid (L–S) interface was found to be orientated in the same direction as the selector channel, and the temperature gradient also altered from 53 °C/cm to 114 °C/cm. As the grain boundary is parallel to the Z-selector channel, the overgrowth rate of the bi-crystal is very low. Because of the combined effect of space expansion and geometric constraint, the initial position of the bi-crystal in the selector has a bigger effect on the overgrowth rate than the effects of the primary and secondary orientations. Changing both the primary and secondary dendritic orientations have a greater impact on grain selection behavior than only changing one of the orientations.

## Figures and Tables

**Table 1:** Some key parameters for solidification modeling [18].

**Initial condition**	
Melting temperature of DD33 superalloy	1550 °C
Mold temperature	1550 °C
Chill-plate temperature	40 °C
**Boundary condition**	
Heater temperature	1550 °C
Emissivity	0.8
Cooler temperature	40 °C
**Interface heat transfer coefficients**	
Alloy melt and ceramic shell mold	200–1900 W (m^2^ K)^−1^
Alloy melt and water-cooled chill plate	1000 W (m^2^ K)^−1^
Ceramic shell mold and water-cooled chill plate	50 W (m^2^ K)^−1^
**Alloy properties**	
Specific heat	358–773 KJ/kg/K
Latent heat	277.4 KJ/kg
Thermal conductivity	6.7–33.2 W/m/K
Density	7.63–8.78 g/cm^3^

**Table 2:** The information of angles and corresponding surface nucleation.

Order Number	Angle between the thin Slice and the YOZ Plane (°)	Surface Nucleation (Single Crystal Type, Euler Angle)					
		Bottom Surface of A			Bottom Surface of B		
		*Φ1* (°)	*Φ* (°)	*Φ2* (°)	*Φ1* (°)	*Φ* (°)	*Φ2* (°)
1	0	0	0	0	0	5	0
2	30	0	0	0	0	5	0
3		0	0	0	0	20	0
4		5	15	0	0	0	0
5		15	15	0	0	0	0
6		45	15	0	0	0	0
7		95	15	0	0	0	0
8		120	15	0	0	0	0
9		150	15	0	0	0	0
10		5	15	5	0	0	0
11		5	15	10	0	0	0
12		5	15	15	0	0	0
13		5	15	25	0	0	0
14		5	15	45	0	0	0
15		5	15	60	0	0	0
16	60	0	0	0	0	5	0
17	90	0	0	0	0	−20	0
18		0	0	0	0	−15	0
19		0	0	0	0	−10	0
20		0	0	0	0	−5	0
21		0	0	0	0	5	0
22		0	0	0	0	10	0
23		0	0	0	0	15	0
24		0	0	0	0	20	0

## Data Availability

Data are contained within the article.
